# Sequential (gemcitabine/vinorelbine) and concurrent (gemcitabine) radiochemotherapy with FDG-PET-based target volume definition in locally advanced non-small cell lung cancer: first results of a phase I/II study

**DOI:** 10.1186/1471-2407-7-112

**Published:** 2007-06-28

**Authors:** Bernd Gagel, Marc Piroth, Michael Pinkawa, Patrick Reinartz, Thomas Krohn, Hans J Kaiser, Sven Stanzel, Christian Breuer, Branka Asadpour, Axel Schmachtenberg, Michael J Eble

**Affiliations:** 1 Department of Radiotherapy, RWTH Aachen University, Germany; 2 Department of Nuclear Medicine, RWTH Aachen University, Germany; 3 Institute of Medical Statistics, RWTH Aachen University, Germany; 4 Department of Internal Medicine, RWTH Aachen University, Germany

## Abstract

**Background:**

The aim of the study was to determine the maximal tolerated dose (MTD) of gemcitabine every two weeks concurrent to radiotherapy, administered during an aggressive program of sequential and simultaneous radiochemotherapy for locally advanced, unresectable non-small cell lung cancer (NSCLC) and to evaluate the efficacy of this regime in a phase II study.

**Methods:**

33 patients with histologically confirmed NSCLC were enrolled in a combined radiochemotherapy protocol. 29 patients were assessable for evaluation of toxicity and tumor response. Treatment included two cycles of induction chemotherapy with gemcitabine (1200 mg/m^2^) and vinorelbine (30 mg/m^2^) at day 1, 8 and 22, 29 followed by concurrent radiotherapy (2.0 Gy/d; total dose 66.0 Gy) and chemotherapy with gemcitabine every two weeks at day 43, 57 and 71. Radiotherapy planning included [^18^F] fluorodeoxyglucose positron emission tomography (FDG PET) based target volume definition. 10 patients were included in the phase I study with an initial gemcitabine dose of 300 mg/m^2^. The dose of gemcitabine was increased in steps of 100 mg/m^2 ^until the MTD was realized.

**Results:**

MTD was defined for the patient group receiving gemcitabine 500 mg/m^2 ^due to grade 2 (next to grade 3) esophagitis in all patients resulting in a mean body weight loss of 5 kg (SD = 1.4 kg), representing 8% of the initial weight. These patients showed persisting dysphagia 3 to 4 weeks after completing radiotherapy. In accordance with expected complications as esophagitis, dysphagia and odynophagia, we defined the MTD at this dose level, although no dose limiting toxicity (DLT) grade 3 was reached.

In the phase I/II median follow-up was 15.7 months (4.1 to 42.6 months). The overall response rate after completion of therapy was 64%. The median overall survival was 19.9 (95% CI: [10.1; 29.7]) months for all eligible patients. The median disease-free survival for all patients was 8.7 (95% CI: [2.7; 14.6]) months.

**Conclusion:**

After induction chemotherapy, the maximum tolerated dose and frequency of gemcitabine was defined at 500 mg/m^2 ^every two weeks in three cycles during a maximum of 7 weeks of thoracic radiotherapy for the phase II study. This regimen represents an effective and tolerable therapy in the treatment of NSCLC.

## Background

In recent years, a number of new, non-platinum agents have demonstrated significant activity in advanced non-small cell lung cancer (NSCLC). These substances include taxanes, vinorelbine and gemcitabine [11]. The novel agents generally have a toxicity profile superior to that of platinum. This fact is highly important in a setting, in which overall quality of life is a major consideration, along with the control of lung cancer symptoms. Combinations of these new drugs in doublets have yielded results which are at least comparable with those achievable with cisplatin-containing regimens in stage IV of NSCLC. The combination of gemcitabine and vinorelbine proved to be particularly feasible; myelosuppression was the most frequent toxicity. Most phase II trials reported response rates of 20–40% and median survival duration of 7–11 months [3,14,15,17].

For locally advanced NSCLC, new treatment approaches utilise both radiotherapy and chemotherapy. Recent data suggest a survival benefit from concomitant radiochemotherapy [1,4,7,16,20], but associated with high percentages of therapy induced side effects, including pneumonitis and esophagitis. The intensity of these toxicities mainly depends on irradiated organ volume.

We conducted a phase I/II study combining gemcitabine with concurrent thoracic radiotherapy in the treatment of patients with locally advanced, unresectable NSCLC. To minimize distant failure and to reduce irradiated volume, we induced all patients with a regimen that consisted of two 21-day cycles of vinorelbine and gemcitabine followed by [^18^F] fluorodeoxyglucose positron emission tomography (FDG PET) based evaluation of tumor response and target volume definition.

In order to determine the maximal tolerated dose (MTD) of gemcitabine every two weeks to a concurrent radiotherapy administered during an aggressive program of sequential and simultaneous radio-/chemotherapy an upstream phase I study was initiated.

## Methods

The Medical Ethics Committee of the University of Aachen approved the phase I/II study. All patients gave written informed consent before they were enrolled in the study. Patient recruitment started in March 2003 and ended in December 2005.

### Patients

Patients with pathologically confirmed NSCLC and clinically evaluated unresectable stage IIIa/b disease were eligible. Patients with a Karnofsky performance status of 80 to 100; age >18 years and forced expiratory volume in 1 second ≥1.2 l were enrolled. Initial laboratory tests included serum creatinine level ≤1.5 mg/dl; granulocyte count ≥2000/ml; platelet count ≥100000/ml; serum bilirubin level ≤1.5 × the normal value; serum glutamic-oxaloacetic transaminase level ≤2.5 × the normal value; and alkaline phosphatase level ≤5 × the normal value. Exclusion criteria were as follows: malignant pleural effusion and/or pericardial effusion; recurrent disease after previous treatment; history of another malignancy; history of anticancer chemotherapy or RT; recent history of myocardial infarction, angina pectoris, congestive heart failure, or uncontrolled arrhythmia within 6 months of diagnosis; pregnancy and missing written informed consent.

### Chemotherapy

The induction chemotherapy consisted of 2 cycles gemcitabine (1200 mg/m^2^) and vinorelbine (30 mg/m^2^) given on day 1, 8 (1.cycle) and on day 22, 29 (2.cycle). Gemcitabine was administered first as a 30-min i.v. infusion, followed by vinorelbine given as a 5-min i.v. infusion.

In the phase I gemcitabine dose ranged between 300 and 500 mg/m^2 ^in 100 mg/m^2 ^increments. MTD was identified for the patient group receiving gemcitabine 500 mg/m^2 ^because of therapy induced esophagitis

### Radiotherapy

The initial irradiated volume included the primary tumor, the ipsilateral hilar lymph node regions and the mediastinal lymph node regions with tumor invasion detected by [^18^F] fluorodeoxyglucose positron emission tomography (FDG PET). The location of lymph node regions within the mediastinum has been described according to the American Thoracic Society's definition (ATS map) [27]. Comparably to De Ruysscher et al, gross tumor volume (GTV) included the postchemotherapy volume of the primary tumor, whereas for the lymph nodes only the pretreatment extension was taken into account [6]. The boost target volume included the primary tumor and any gross adenopathy detected by post chemotherapy FDG PET. To ensure accurate and reproducible positioning setups, PET and planning CT scans were performed using an immobilization device. For the first patients target volumes were delineated on planning computed tomography (CT) scans by a visual fusion technique [27], followed by respiratory gated CT scans as well as rigid co-registration of PET and CT using a normalised mutual information algorithm (Hermes^®^; Nuclear Diagnostics, Stockholm, Sweden). Optimizing PET and CT fusion, CT scans in exspiration were used. Radiotherapy was administrated with 10- or 15-MV photons. Radiation was delivered in 2-Gy fractions daily for 5 days per week to a total dose of 50 Gy for the initial volume. The boost target volume, received additional 16 Gy in the same dose fractionation. Isodose curves and dose-volume histograms (DVHs) were calculated for the lungs, the esophagus, the heart and the spinal cord. The maximum dose to any level of the spinal cord did not exceed 45 Gy.

### Dose modification

Dose modification of the gemcitabine and vinorelbine application (induction chemotherapy) was specified for myelosuppression and hepatic dysfunction, including a therapy delay of 1 week. No dose reductions were allowed in phase I of the study.

### Assessment of response and toxicity

Toxicity was assessed using the CTC scale modified by AIO/ARO/ADT. MTD was defined as the highest safely tolerated dose with toxicity levels that did not exceed any of the following DLTs at one dose level: grade III hematologic toxicities (≤ 2 of 6 patients); grade III cardiac or pulmonary toxicity (≤ 2 of 6 patients); grade III esophagitis (≤ 2 of 6 patients) and other unexpected, relevant grade III toxicities (≤ 2 of 6 patients). The initial dose was given to the first three patients. If none of these patients experienced a DLT, then dose escalation proceeded to the next level. If one of the first three patients experienced a DLT, enrolment was continued until six patients were enrolled, or a third DLT occurred. If the number of DLTs was ≤ 2, dose escalation continued, otherwise dose escalation was stopped.

Staging was done in accordance with the International Staging System [18]. Pretreatment evaluation included patient history, physical examination, routine laboratory studies, computed tomography scans of the thorax, and abdomen. Bone scans and FDG-PET were required initially. Pulmonary function was evaluated before induction therapy, 2 weeks after completion of the chemotherapy and 6 weeks after the chemoradiotherapy phase. Patients were monitored in weekly intervals for adverse events by taking history, physical examination, laboratory studies, and toxicity assessment during the induction and consolidation treatment. Acute toxicity was scored from the start to 3 months after the end of radiotherapy. Late toxicity was scored more than 3 months after the end of radiotherapy.

Restaging was performed 10 to 14 days after completion of induction chemotherapy and 6 weeks after completion of consolidation radiochemotherapy. Follow-up evaluation consisted of medical history and physical examination with examinations of blood pressure and heart rate followed by electrocardiogram and heart ultrasound in the case of hypertension or irregular heart beat. In addition imaging studies including CT scans of the thorax and abdomen as well as FDG-PET (for the first two follow-up evaluations) or every 3 months for 18 months and every 6 months thereafter were performed. Tumor response was evaluated by CT data according to response evaluation criteria in solid tumors (RECIST). In the case of CT based residual tumor without pathologic FDG uptake, complete remission was defined.

### Statistical methods

Categorical data were summarized by absolute and/or relative frequencies. Furthermore, 95% confidence intervals for therapy response rates were computed according to the method of Pearson and Clopper [5].

Patient follow-up times were condensed by median, minimum and maximum value, whereas the arithmetric mean and the corresponding standard deviation (SD) were used for data of all other continuous variables.

Observed values of various lung function parameters six weeks respectively six months after therapy were compared to corresponding baseline values using paired t-tests on a global significance level of α = 0.05. The Bonferroni method was used for multiple testing adjustment of the p-values obtained from the 18 paired t-tests conducted; adjusted p-values of p ≤ 0.05 can be interpreted as statistically significant test results.

Overall as well as disease-free survival times of the patients were investigated by Kaplan-Meier survival analysis. Moreover, median overall as well as disease-free survival times and corresponding 95% confidence intervals were calculated.

All confidence intervals computed were interpreted in a solely descriptive manner. All statistical analyses were performed using the SPSS^® ^statistical analysis software package, version 12.0.

## Results

### Patients

A total of 33 patients were enrolled in this study from March 2003 to December 2005: Ten patients onto the phase I component and additional 23 patients onto the phase II study. 29 patients were assessable for evaluation of toxicity and response. One patient refused the second cycle of chemotherapy after signing consent. Three patients were excluded from the study because of early deterioration and death: acute myocardial infarction (1), lung embolism (1) and histologically proven atypical pneumonia caused by cytomegalovirus without leucopenia or neutropenia during the treatment (1). Patient characteristics are listed in Table 1.

**Table 1:** Characteristics of population

	Dose Level		
	
	300 mg/m^2^	400 mg/m^2^	500 mg/m^2^
No. of patients	3	3	23
Age (years)			
Mean	68	50	58
Range	63–75	42–62	40–75
Sex			
Male	3	1	17
Female	0	2	6
Karnofsky Performance Status			
100%	2	1	9
90%	0	1	7
80%	1	1	7
Clinical stage			
cT2cN3	0	0	2
cT3cN2	2	0	1
cT3cN3	0	0	6
cT4 cN0	0	0	6
cT4 cN2	0	2	5
cT4 cN3	1	1	3
Histology			
Adenocarcinoma	0	2	6
Squamous Carcinoma	1	1	15
Adenosquamous Carcinoma	1	0	1
Large-Cell Carcinoma	1	0	1

### Radiotherapy

The heart was irradiated with a mean dose of 11.05 Gy (SD = 7.01 Gy). The mean applied total lung dose was 12.31 Gy (SD = 2.64 Gy) based on a mean ipsilateral lung dose of 19.85 Gy (SD = 4.03 Gy) and a mean contralateral lung dose of 6.52 Gy (SD = 3.34 Gy). The mean ratio between contralateral to ipsilateral lung volume was 1.22 with a mean ipsilateral lung volume of 1918.4 cm^3 ^(SD = 679.4 cm^3^), and a mean contralateral lung volume of 2332.1 cm^3 ^(SD = 556.3 cm^3^). The esophagus (mean volume = 37.8 cm^3^, SD = 10.6 cm^3^) was irradiated with a mean dose of 29.0 Gy (SD = 6.4 Gy). The mean initial planning target volume was 735.0 cm^3 ^(SD = 266.7 cm^3^); the mean boost volume was 399.7 cm^3 ^(SD = 239.2 cm^3^). Different dosimetric parameters for lung exposure are listed in Table 2.

**Table 2:** Descriptive summary of dosimetric parameters for lung exposure

	Total Lung		Ipsilat. Lung		Contralat. Lung	
			
	Mean	SD	Mean	SD	Mean	SD
Mean Lung Dose	12.31	2.64	19.85	4.03	6.52	3.34
V20	26.77	7.61	44.64	9.87	13.55	8.85
V30	20.26	5.47	36.00	6.51	8.55	6.66
V40	15.93	5.01	30.00	5.50	5.59	4.99

### Toxicity

Toxicity caused by induction chemotherapy was predictable and acceptable. Only nonfebrile leucocytopenia and clinically insignificant anaemia were observed. Four patients (14%) of the phase II required treatment delays during sequential chemotherapy caused by poststenotic pneumonia without leucocytopenia or neutropenia. Because of treatment delay and the necessity of effective local tumor treatment, in three of these patients concurrent radiochemotherapy was started after only 3 applications of gemcitabine and vinorelbine. One patient showed cardial symptoms including tachycardia and thoracic pain after application of vinorelbine. Consequently gemcitabine was not infused. Representing a unique effect without pathologic findings in cardial examination, the subsequent chemotherapy could be applied routinely. Analysing late cardiac side effects, one patient showed minimal pericardial effusion being treated with a diuretic. No significant change in blood pressure or irregular heart beat was diagnosed.

Significant esophageal and pulmonary toxicities were encountered as a result of concurrent treatment. In the third group (500 mg/m^2^) of the phase I, grade 2 esophagitis was encountered in all four patients. In contrast to the second group receiving 400 mg/m^2^, where two patients showed grade 2 esophagitis including moderate dysphagia, all 4 patients in the third group showed severe dysphagia making intensified local treatment necessary. They showed a mean body weight loss of 5 kg (SD = 1.4 kg) representing 8% of the initial weight. Furthermore these patients showed persisting dysphagia 3 to 4 weeks after completing radiotherapy. Three months after finishing therapy none of the four patients described persisting dysphagia.

Because of the described severe increase in esophagitis in the third group and the expected complications such as severe dysphagia and odynophagia, ulceration or occlusion, we did not realize the next step of dose escalation. We defined the MTD at a dose level of 500 mg/m^2 ^every two weeks, although no dose-limiting toxicity grade 3 according to CTC criteria was reached [9].

In the phase II study, 7 patients showed grade 3 esophagitis (30%), two patients required treatment delays. One of these patients developed hemoragic esophagitis resulting in a 10 day treatment delay whereas in the other case treatment could be continued after a 6 day break. No dose-limiting esophagitis was observed. None of the patients required gastrostomy tubes or esophageal dilation. Prematurely termination of radiochemotherapy after 58.0 Gy was necessary in two patients of the phase II study. One patient showed tumor progression during radiochemotherapy including local progression as well as pulmonary metastases. The other patient developed dose-limiting acute pulmonary toxicity (grade 3), presented with fever, dyspnea, and infiltrates.

Four patients in the phase II developed reversible grade 3 pulmonary toxicity one to six weeks after finishing radiotherapy with local advanced pulmonary infiltrates in CT scans. According to clinical pulmonary symptoms, all patients were treated with steroids and antibiotic i.v. as well as oxygen, reaching adequate stabilization of their symptoms within one week. Toxicities during induction chemotherapy and radiochemotherapy are summarized in Table 3.

**Table 3:** Events of toxicity during chemo- and radiochemotherapy (* = phase I study)

		Esophageal			Pulmonary			Hematologic		
		G1	G2	G3	G1	G2	G3	G1	G2	G3
Gemcitabine (mg/m^2^)	No.	3	19	7	18	6	5	12	9	1
300*	3	1	2	0	1	2	0	1	1	0
400*	3	1	2	0	1	2	0	1	2	0
500*	4	0	4	0	4	0	0	0	1	0
500	19	1	11	7	12	2	5	10	5	1

One patient of the phase II developed cough and dyspnea three weeks after radiochemotherapy. Radiographic pulmonary infiltrates according to an atypical pneumonia were diagnosed. These infiltrates were distributed symmetrically in both lobes of the lung. There was no accordance to the irradiation fields, making radiation induced pneumonia improbable. The patient died of progressive respiratory failure. Refusing post-mortem examination it was impossible to clarify the exact causes of pneumonia.

In order to detect therapy induced side effects, lung function was tested in 25 patients six weeks and in 20 patients six months after radiochemotherapy, excluding patients with progressive disease in the lung. The development in lung function parameters and the mean differences six weeks and six months after finishing radiochemotherapy are listed in Table 4. No significant changes in lung function parameters were found.

**Table 4:** Descriptive summary of baseline values of various lung function parameters as well as of differences in those variables between baseline values and values obtained 6 weeks respectively 6 months after radiochemotherapy (RT/CHT.); corresponding p-values were calculated by conducting paired t-tests assessing whether those differences deviate statistically significant from zero.

	Baseline		6 Weeks after RT/CHT vs Baseline			6 Months after RT/CHT vs Baseline		
			
	Mean	SD	Mean Diff.	SD	p-value	Mean Diff.	SD	p-value
FVC (L)	3.25	0.83	-0.01	0.55	1.00	-0.18	0.45	1.00
FEV1 (L)	2.16	0.70	-0.02	0.32	1.00	-0.14	0.28	0.72
FEV1/VC (%)	63.62	15.38	2.86	17.06	1.00	2.91	14.16	1.00
RV (L)	3.01	0.82	-0.30	0.47	0.16	-0.11	0.71	1.00
TLC (L)	6.25	1.22	-0.33	0.72	0.54	-0.29	0.71	1.00
RV/TLC (%)	47.91	7.76	-2.35	5.85	1.00	-0.42	7.19	1.00
KCO (ml/(min*mmHg*L))	5.74	2.26	-0.34	1.49	1.00	0.08	2.32	1.00
Pao_2 _(mmHg)	62.89	6.40	2.61	6.24	1.00	0.27	6.53	1.00
Paco_2 _(mmHg)	39.12	2.71	-2.01	3.97	0.54	-1.47	3.70	1.00

### Tumor response

**Figure 1:**
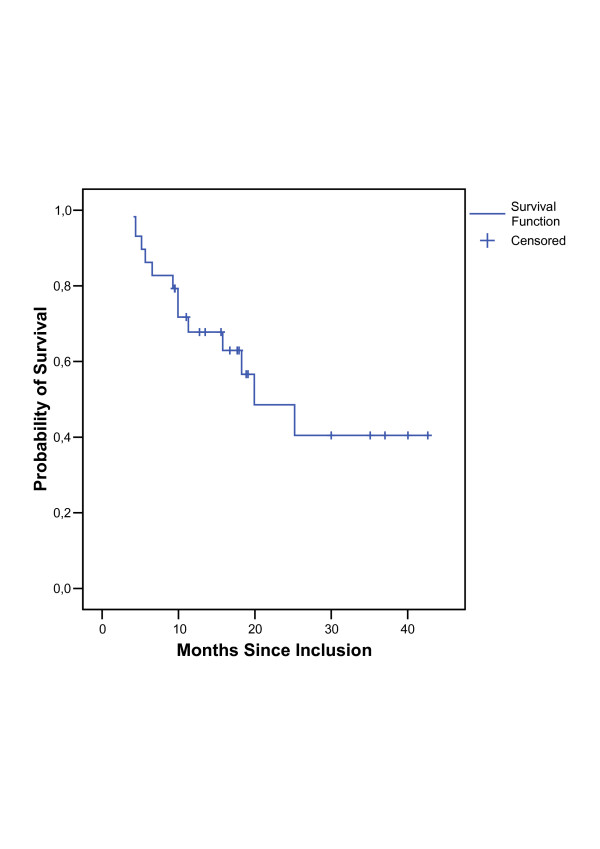
Distribution of overall survival.

**Figure 2:**
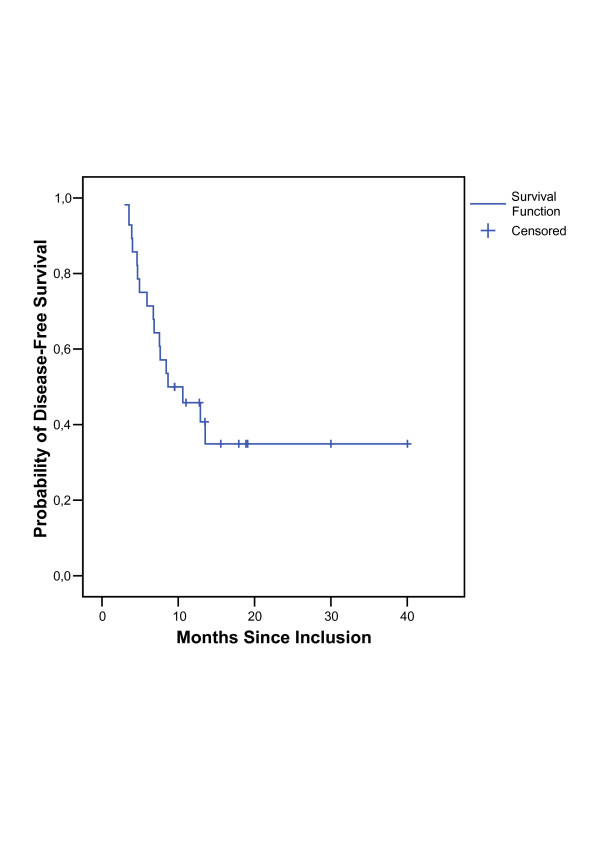
Distribution of disease-free survival.

Median overall follow-up was 15.7 months (4.1 to 42.6 months), the mean observation time in survivors was 21.7 months (5.9 to 42.6 months). Tumor regression after induction chemotherapy could be diagnosed in 19 assessable patients. None of the patients developed progressive disease (distant metastases or local tumor progression). After radiochemotherapy 11 patients reached a complete remission. The local overall response rate (including response of primary tumor and metastatic lymph nodes) after completion of therapy was 83% (95% CI: [64%; 94%]). Analysing local tumor response, 13 patients showed a local partial remission, 2 patients a local stable disease and 3 patients a local progressive disease. 8 of these 18 patients without complete remission still had metastases in PET at the first follow up resulting in an overall response rate of 62% (95% CI: [42%; 79%]). The median overall survival was 19.9 (95% CI: [10.1; 29.7]) months for all eligible patients (Figure 1). The median disease-free survival for all patients was 8.7 (95% CI: [2.7; 14.6]) months (Figure 2). Table 5 shows the response rates six to twelve weeks after radiochemotherapy. In Table 6 the sites of initial relapse are listed.

**Table 5:** Local tumor response and overall tumor response six to twelve weeks after treatment

		Local Response			
		
After Radiochemotherapy		CR	PR	SD	PD
Gemcitabine (mg/m^2^)	No. of Patients				

300	3	1	2	-	-
400	3	1	1	-	1
500	23	9	10	2	2

		Overall Response			
		
After Radiochemotherapy		CR	PR	SD	PD

300	3	1	1	-	1
400	3	1	0	-	2
500	23	9	6	2	6

CR = complete remission, PR = partial remission, SD = stable disease, PD = progressive disease

**Table 6:** Sites of initial relapse

Site of tumor progression	Cohort		
	
	300 mg/m^2^	400 mg/m^2^	500 mg/m^2^
	
*No. of Patients*	*3*	*3*	*23*
Locoregional – Infield	2	1	8
Locoregional – Outfield	2	-	-
Locoregional – Infield and Distant	1	-	3
Locoregional – Outfield and Distant	-	-	-
Distant only	-	1	4
Brain	-	-	3
Death without Progression	-	-	1

Discrepancy between disease-free survival and overall survival was caused by three patients. Five months after reaching complete remission one patient developed an increase of bilateral hilar lymph nodes in CT as well as an increase of mean SUV up to 6.4. Second line chemotherapy (carboplatin/paclitaxel) was started without histological verification of malignity. 12 months after second line treatment, there was still a stable SUV of 2.7 at the hilar lymph nodes with stable extension in CT. One patient showed stable partial remission in CT and stable local mean SUV of 3.1 to 3.8 for a time interval of 13 months after radiochemotherapy. In another patient single metastasis of the right suprarenal gland was detected. Receiving stereotactic irradiation with 5 × 8.0 Gy (80% isodose) this patient reached complete remission for the last 23 months.

## Discussion

The use of induction chemotherapy has been developed through a series of clinical trials since the mid-1980s. The Cancer and Leukemia Group B (CALGB) 8433 trial demonstrated significant improvements in the combined modality therapy group, with a longer median survival time (13.7 months vs. 9.6 months; p = 0.012) and higher 5-year survival rate of 17% versus 6% [7].

Several phase III trials compared concurrent radiochemotherapy with radiotherapy alone. The first of these trials was performed by the European Organization for Research and Treatment of Cancer (EORTC). In that trial, 331 patients were randomized to three treatment programs [21]. The 3-year survival rate was significantly greater with the daily application of cisplatin (arm 3) than with the thoracic radiotherapy alone (arm1) (16% vs. 2%; p = 0.009). The survival benefit of weekly cisplatin (arm 2) was intermediate and not statistically different from both groups. The time to local recurrence was significantly longer in the cisplatin groups (p = 0.015), especially the group with cisplatin daily (p = 0.003).

The consequence was the conduction of several trials comparing systemic chemotherapy plus concurrent radiotherapy with several cycles of systemic chemotherapy followed by radiotherapy (sequential approach). In the NPC 95-01 study patients were randomly assigned to receive sequential or concurrent therapy [8]. In the sequential arm, three cycles of chemotherapy were administered first, consisting of cisplatin and vinorelbine, followed by thoracic RT at a dose of 66 Gy in 33 fractions (2 Gy per fraction, 5 fractions per week) at day 78. In the concurrent arm, the same radiotherapy was started on day 1 with two concurrent cycles of cisplatin and etoposide (days 1 to 5 and days 29 to 33). The patients received two cycles consolidation therapy with cisplatin and vinorelbine afterwards. The median survival was 14.5 (95% CI: [8.3; 27.4]) months in the sequential arm and 16.3 (95% CI: [5.8; 34.8]) months in the concurrent treatment. Nevertheless this difference was not significant. Esophageal toxicity was significantly more frequent in the concurrent arm than in the sequential arm (32% vs. 3%). Analysing treatment protocol 88% of the patients in the concurrent arm but only 60% in the sequential arm received an irradiation dose higher than 60 Gy. This imbalance in radiotherapy was also described by Zatloukal et al, comparing concurrent versus sequential radiochemotherapy with cisplatin and vinorelbine in a randomised trial [28].

In our study we supplied gemcitabine and vinorelbine as dose intense induction chemotherapy in a short time interval of two weeks, in order to kill malignant cells in existing micro-metastases and to reduce the planning target volume (PTV). The latter could be realized in 19 of 29 patients receiving a tumor regression after induction chemotherapy. Recent studies in patients with solid tumors have shown it's value as a radiotherapy enhancer, even at doses substantially below those required for cytotoxic effects [23-26]. Therefore we used gemcitabine as a radiotherapy enhancer in a dose intense treatment to improve local tumor control. On the basis of a possible accumulation of side effects after intensified induction chemotherapy, a dose-finding study was carried out to minimize radiation-induced complications. Furthermore, gemcitabine was administered only once every two weeks, concurrent to irradiation to avoid dose reduction in radiotherapy. In the phase II, only two patients needed premature termination of radiochemotherapy after 58.0 Gy. One patient showed tumor progression during radiochemotherapy, the other patient developed dose-limiting acute pulmonary toxicity (grade 3). Because of reduced lung function in combination with high lung exposure in six patients (21%) the applied total dose was reduced to 60.0 Gy.

In the phase I, dose-limiting toxicity (DLT) was identified for the patient group receiving gemcitabine 500 mg/m^2^, due to grade 2 esophagitis (next to grade 3) in all patients. In accordance with expected complications, such as esophagitis, dysphagia and odynophagia, we defined the MTD at this dose level although no DLT grade 3 occurred.

We already described the problem of scoring toxicity by clinical symptoms because of different scores and interinstitutional ambiguity in the scoring of esophagitis cases for lung cancer patients, as well as differences in policy to prescribe analgetics or i.v. fluids and hyperalimentation (indicative for grade 3–4 esophagitis) [9]. According to CTC criteria modified by AIO/ARO/ADT, grade 3 toxicity was defined as very painful dysphagia, oedema or ulceration with necessity of pure/liquid diet or analgetics. In contrast to the phase I study where we avoided systemic analgetics, we used it in the phase II study in combination with supportive hyperalimentation(per definition grade 3 toxicity) resulting in grade 3 esophagitis in 37% of the patients. Consequently only two of these patients required treatment delays with one patient developing hemoragic esophagitis. No dose-limiting esophagitis was developed.

In the phase II study five patients developed grade 3 pulmonary toxicities. Based on CT scans and lung function testing in four of these patients, advanced pulmonary emphysema based on chronic obstructive pulmonary disease was diagnosed, increasing the risk of radiation induced pneumonia [19]. All patients were treated with steroids and antibiotics i.v. as well as oxygen, reaching adequate stabilization and relief of their symptoms within one to two weeks. Analysing the different lung parameters, we found only a slight decrease of FEV1 of at mean 0.14 l.

In a prospective study, Kalff et al found that FDG PET scanning changed or influenced management decisions in 67% of patients with NSCLC [13]. Patients were frequently spared unnecessary treatment, and management was more appropriately targeted. According to radiotherapy, PET influenced the intent, modality, or delivery of treatment in 36 (64%) of 56 patients, as compared to a prospectively recorded pre-PET plan. In our study, additional organ metastases were diagnosed by the use of FDG-PET in four patients that had not been detected in CT scans of the thorax and abdomen.

Analysing FDG-PET in problematic mediastinal lymph node staging, a sensitivity of 80% and specificity of 88% can be expected [12]. In addition, a good correlation between histological regression grades after radiochemotherapy and metabolic remission as detected by PET could be shown [22]. If chemotherapy is effective as a systemic adjuvant therapy, it should also enable to control occult microscopic nodal disease, obviating the need of elective nodal irradiation [2]. Therefore we used the initial FDG PET for definition of nodal irradiation, including the primary involved lymph node regions or the regions with high risk of tumor invasion. After 50.0 Gy, only the gross tumor volume, as identified after induction chemotherapy in the second FDG PET, was irradiated resulting in a target volume reduction of 335.3 cm^3 ^(SD = 239.2 cm^3^). In order to emphasize the effectiveness of PET based target volume definition, local tumor response was evaluated. Only in two patients (7%) locoregional outfield relapses were observed.

In the future, biological images which detect probable resistance to therapy, such as hypoxic tumor regions, i.e. through the use of [^18^F] fluoromisonidazole positron emission tomography (FMISO-PET), may provide information for defining a biological target volume (BTV) to improve local control and simultaneously reduce the exposure of organs at risk [10].

## Conclusion

Gemcitabine and vinorelbine as induction chemotherapy, followed by gemcitabine concurrent with thoracic radiotherapy every two weeks is feasible. Esophagitis was found to be the DLT of gemcitabine with thoracic irradiation. Hematological side effects were moderate. We recommend a gemcitabine dose of 500 mg/m^2 ^every two weeks, when administered with thoracic radiotherapy for 6 to 7 weeks. Although there was increase in esophageal and pulmonal toxicity in phase II, this treatment modality is well tolerated. Because of favourable survival and acceptable toxicity profile, we consider this radiochemotherapy regime combined with PET based target volume definition as a warrant for further evaluation.

## Competing interests

Financial competing interests

The pharmaceutical company Hoffmann-La Roche partially financed the costs for FMISO, a substance used to carry out PET scans.

The authors declare that there are no other competing interests.

## Authors' contributions

BG has made substantial contributions to conception and design, acquisition of data, analysis and interpretation of data; MP has been involved in acquisition of data; MP has been involved in acquisition of data; PR has made substantial contributions to conception and design, acquisition of data, analysis and interpretation of data; TK has been involved in acquisition of data, analysis and interpretation of data; HJK has been involved in acquisition of data, analysis and interpretation of data; SS has been involved in analysis and interpretation of data; CB has been involved in acquisition of data; BA has been involved in acquisition of data; AS has been involved in analysis and interpretation of data; MJE has been involved in analysis and interpretation of data and has made contributions to conception and design.

All authors read and approved the final manuscript.

## Pre-publication history

The pre-publication history for this paper can be accessed here:



## References

[B1] Albain KS, Crowley JJ, Turrisi AT, Gandara DR, Farrar WB, Clark JI, Beasley KR, Livingston RB (2002). Concurrent cisplatin, etoposide, and chest radiotherapy in pathologic stage IIIB non-small-cell lung cancer: A Southwest Oncology Group phase II study, SWOG 9019. J Clin Oncol.

[B2] Armstrong JG (1998). Target volume definition for three-dimensional conformal radiation therapy of lung cancer. Br J Radiol.

[B3] Chen YM, Perng RP, Yang KY, Liu TW, Tsai CM, Ming-Liu J, Whang-Peng J (2000). A multicenter phase II trial of vinorelbine plus gemcitabine in previously untreated inoperable (stage IIIB/IV) non-small cell lung cancer. Chest.

[B4] Choy H, Akerley W, Safran H, Graziano S, Chung C, Williams T, Cole B, Kennedy T (1998). Multi-institutional phase II trial of paclitaxel, carboplatin, and concurrent radiation therapy for locally advanced non-small-cell lung cancer. J Clin Oncol.

[B5] Clopper CJ, Pearson ES (1934). The use of confidence or fiducial limits illustrated in the case of the binomial. Biometrika.

[B6] De Ruysscher D, Wanders S, Minken A, Lumens A, Schiffelers J, Stultiens C, Halders S, Boersma L, Baardwijk A, Verschueren T, Hochstenbag M, Snoep G, Wouters B, Nijsten S, Bentzen SM, Kroonenburgh M, Ollers M, Lambin P (2005). Effects of radiotherapy planning with a dedicated combined PET-CT-simulator of patients with non-small cell lung cancer on dose limiting normal tissues and radiation dose-escalation: A planning study. Radiother Oncol.

[B7] Dillman RO, Herndon J, Seagren SL, Eaton WL, Green MR (1996). Improved survival in stage III non-small-cell lung cancer: seven-year follow-up of Cancer and Leukemia Group B (CALGB) 8433 trial. J Natl Cancer Inst.

[B8] Fournel P, Robinet G, Thomas P, Souquet PJ, Lena H, Vergnenegre A, Delhoume JY, Le Treut J, Silvani JA, Dansin E, Bozonnat MC, Daures JP, Mornex F, Perol M (2005). Randomized phase III trail of sequential chemoradiotherapy compared with concurrent chemoradiotherapy in locally advanced non-small cell lung cancer: Group Lyon-Saint Etienne d'Onncologie Thoracique – Group Francaise de Pneumo-Cancerologie NPC 95-01 Study. J Clin Oncol.

[B9] Gagel B, Piroth M, Michael Pinkawa M, Patrick Reinartz P, Zimny M, Fischedik K, Stanzel S, Breuer C, Skobel E, Asadpour B, Schmachtenberg A, Buell U, Eble MJ (2006). Gemcitabine Concurrent with Thoracic Radiotherapy after Induction Chemotherapy with Gemcitabine/Vinorelbine in Locally Advanced Non-Small Cell Lung Cancer. Strahlenther Onkol.

[B10] Gagel B, Reinartz P, Demirel C, Kaiser HJ, Zimny M, Piroth M, Pinkawa M, Stanzel S, Asadpour B, Hamacher K, Coenen HH, Buell U, Eble MJ (2006). [18F]fluoromisonidazole and [18F] fluorodeoxyglucose positron emission tomography in response evaluation after chemo-/radiotherapy of non-small-cell lung cancer: a feasibility study. BMC Cancer.

[B11] Giaccone G (1996). New drugs for the management of lung cancer. Br J Hosp Med.

[B12] Gould MK, Kuschner WG, Rydzak CE, Maclean CC, Demas AN, Shigemitsu H, Chan JK, Owens DK (2003). Test performance of positron emission tomography and computed tomography for mediastinal staging in patients with non-small cell lung cancer: A meta-analysis. Ann Intern Med.

[B13] Kalff V, Hicks RJ, MacManus P, Binns DS, McKenzie AF, Ware RE, Hogg A, Ball DL (2001). Clinical impact of [^18^F]fluorodeoxyglucose positron emission tomography in patients with non-small-cell lung cancer: a prospective study. J Clin Oncol.

[B14] Krajnik G, Mohn-Staudner A, Thaler J, Greil R, Schmeikal S, Marhold F, Deutsch J, Preiss P, Malayeri R, Schafer-Prokop C, Wein W, Huber H, Pirker R (2000). Vinorelbine-gemcitabine in advanced non-small cell lung cancer (NSCLC): an AASLC phase II trial. Ann Oncol.

[B15] Laack E, Mende T, Benk J, Chemaissani A, Scholtze J, Lorenz C, Niestroy A, Dalhoff K, Muller T, Walter T, Durk H, Edler L, Hossfeld DK (2001). Gemcitabine and vinorelbine as first-line chemotherapy for advanced non-small cell lung cancer, a phase II trial. Eur J Cancer.

[B16] Le Chevalier T, Arriagada R, Quoix E, Ruffie P, Martin M, Tarayre M, Lacombe-Terrier MJ, Douillard JY, Laplanche A (1991). Radiotherapy alone versus combined chemotherapy and radiotherapy in nonresectable non-small-cell lung cancer: First analysis of a randomized trial in 353 patients. J Natl Cancer Inst.

[B17] Lorusso V, Carpagnano F, Frasci G, Panza N, Di Rienzo G, Cisternino ML, Napoli G, Orlando S, Cinieri S, Brunetti C, Palazzo S, De Lena M (2000). Phase I/II study of gemcitabine plus vinorelbine as first-line chemotherapy of non-small-cell lung cancer. J Clin Oncol.

[B18] Mountain CF (1997). Revisions in the international system for staging lung cancer. Chest.

[B19] Rancati T, Ceresoli GL, Gagliardi G, Schipani S, Cattaneo GM (2003). Factors predicting radiation pneumonitis in lung cancer patients: a retrospective study. Radiother Oncol.

[B20] Sause WT, Scott C, Taylor S, Johnson D, Livingston R, Komaki R, Emami B, Curran WJ, Byhardt RW, Turrisi AT, Dar AR, Cox JD (1995). Radiation Therapy Oncology Group (RTOG) 88-08 and Eastern Cooperative Oncology Group (ECOG) 4588: Preliminary results of a phase III trial in regionally advanced, unresectable non-small-cell lung cancer. J Natl Cancer Inst.

[B21] Schaake-Koning C, van den Bogaert W, Festen DO, Hoogenhout J, van Houtte P, Kirkpatrick A, Koolen M, Maat B, Nijs A (1992). Effects of concomitant cisplatin and radiotherapy on inoperable non-small-cell lung cancer. N Engl J Med.

[B22] Schmücking M, Baum RP, Bonnet R, Junker K, Müller K-M (2005). Correlation of histologic results with PET findings for tumor regression and survival in locally advanced non-small cell lung cancer after neoadjuvant treatment. Pathologe.

[B23] Shewach DS, Hahn TM, Chang E, Hertel LW, Lawrence TS (1994). Metabolism of 2',2'difluoro-2'-deoxycytidine and radiation sensitization of human colon carcinoma cells. Cancer Res.

[B24] Shewach DS, Lawrence TS (1996). Radiosensitization of human solid tumor cell lines with gemcitabine. Semin Oncol.

[B25] Trodella L, Granone P, Valente S, Turriziani A, Macis G, Corbo GM, Margaritora S, Cesario A, D'Angelillo RM, Gualano G, Ramella S, Galetta D, Cellini N (2002). Phase I Trial of Weekly Gemcitabine and Concurrent Radiotherapy in Patients with Inoperable Non-Small-Cell Lung Cancer. J Clin Oncol.

[B26] Van Putten J, Price A, van der Leest A, Gregor A, Little FA, Groen HJM (2003). A Phase I Study of Gemcitabine with Concurrent Radiotherapy in Stage III, Locally Advanced Non-Small Cell Lung Cancer. Clinical Cancer Research.

[B27] Vansteenkiste JF, Stroobants SG, Dupont PJ, De Leyn PR, De Wever WF, Verbeken EK, Nuyts JL, Maes FP, Bogaert JG (1998). FDG-PET scan in potentially operable non-small cell lung cancer: do anatometabolic PET-CT fusion images improve the localisation of regional lymph node metastases? The Leuven Lung Cancer Group. Eur J Nucl Med.

[B28] Zatloukal P, Petruzelka L, Zemanova M, Havel L, Janku F, Judas L, Krepela E, Fiala P, Pecen L (2004). Concurrent versus sequential radiochemotherapy with cisplatin and vinorelbine in locally advanced non-small cell lung cancer. Lung Cancer.

